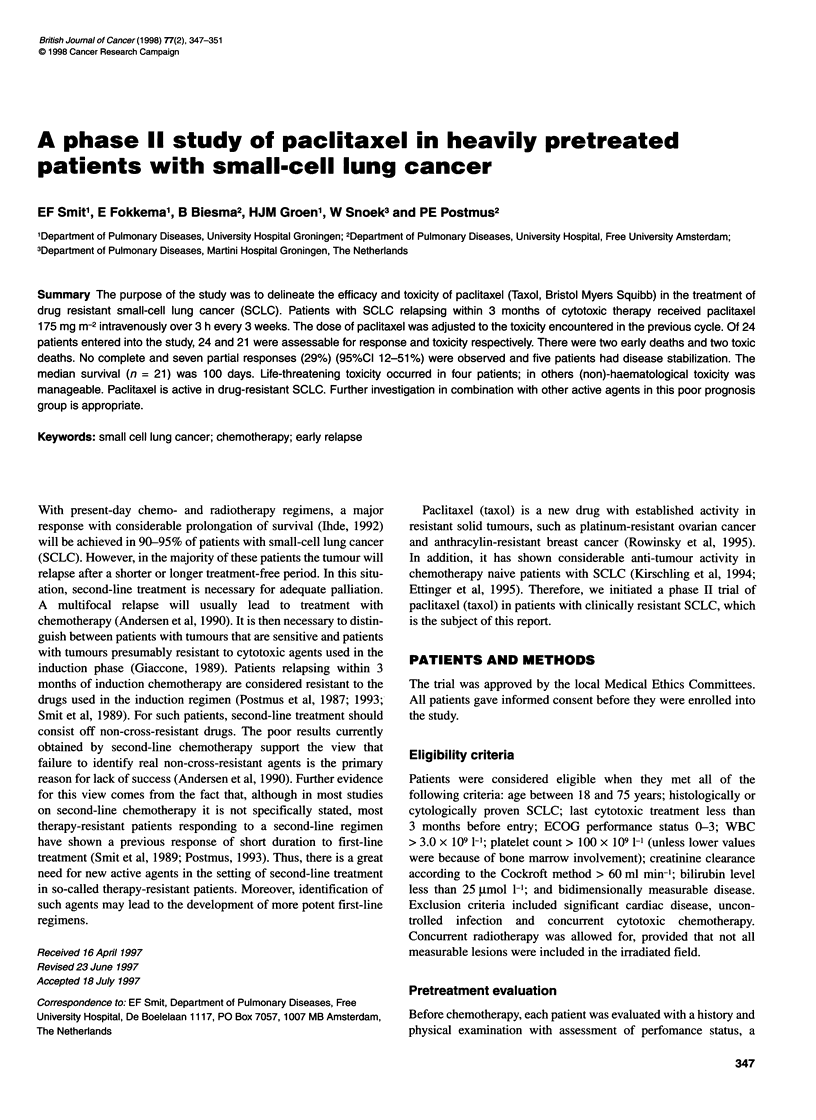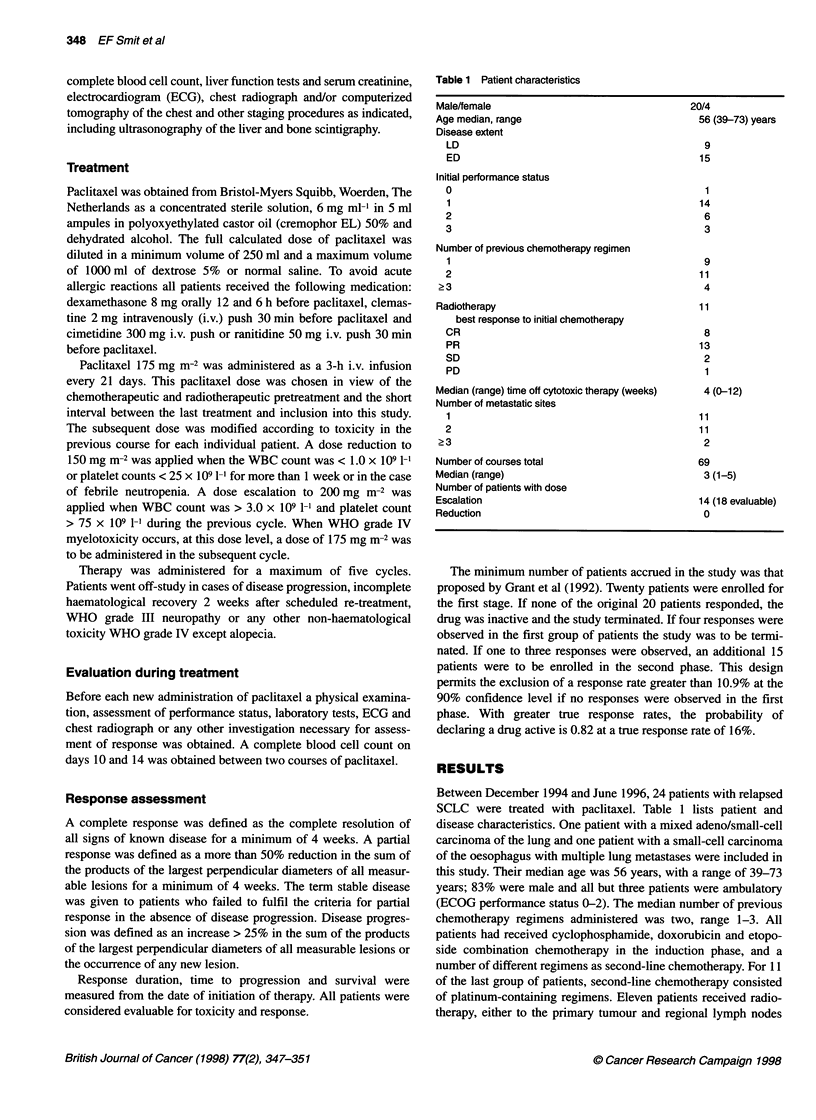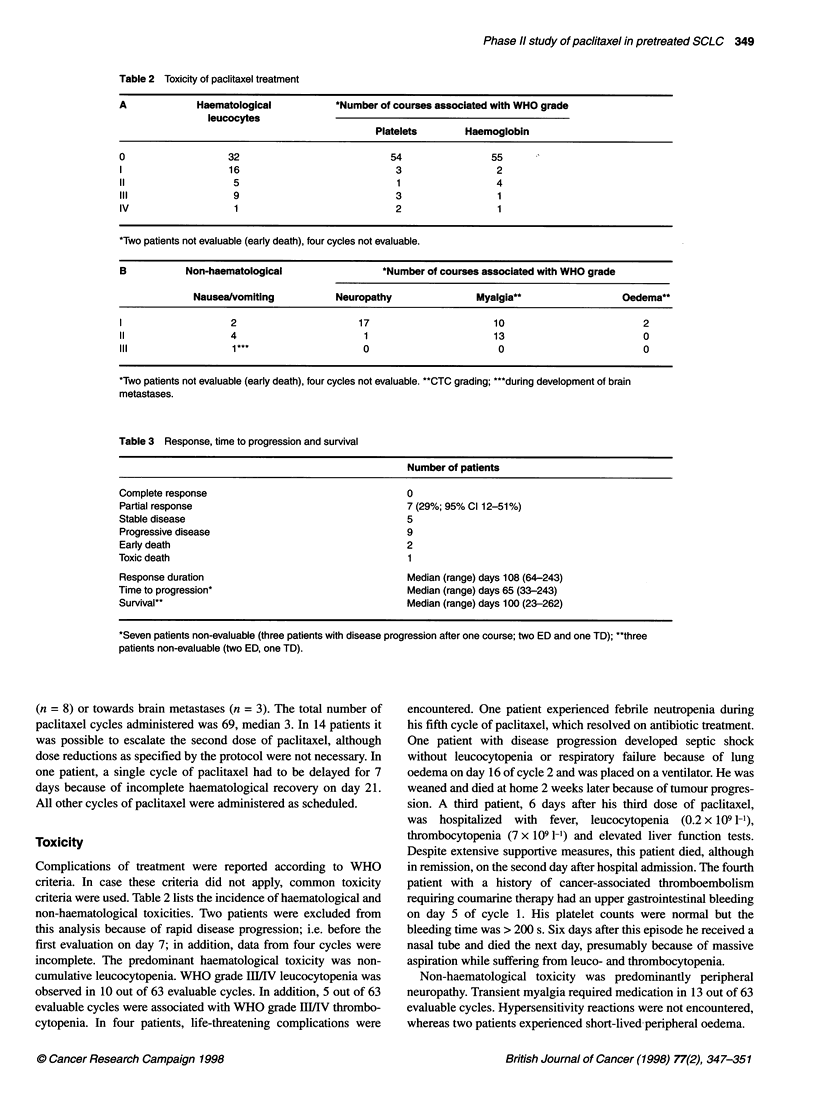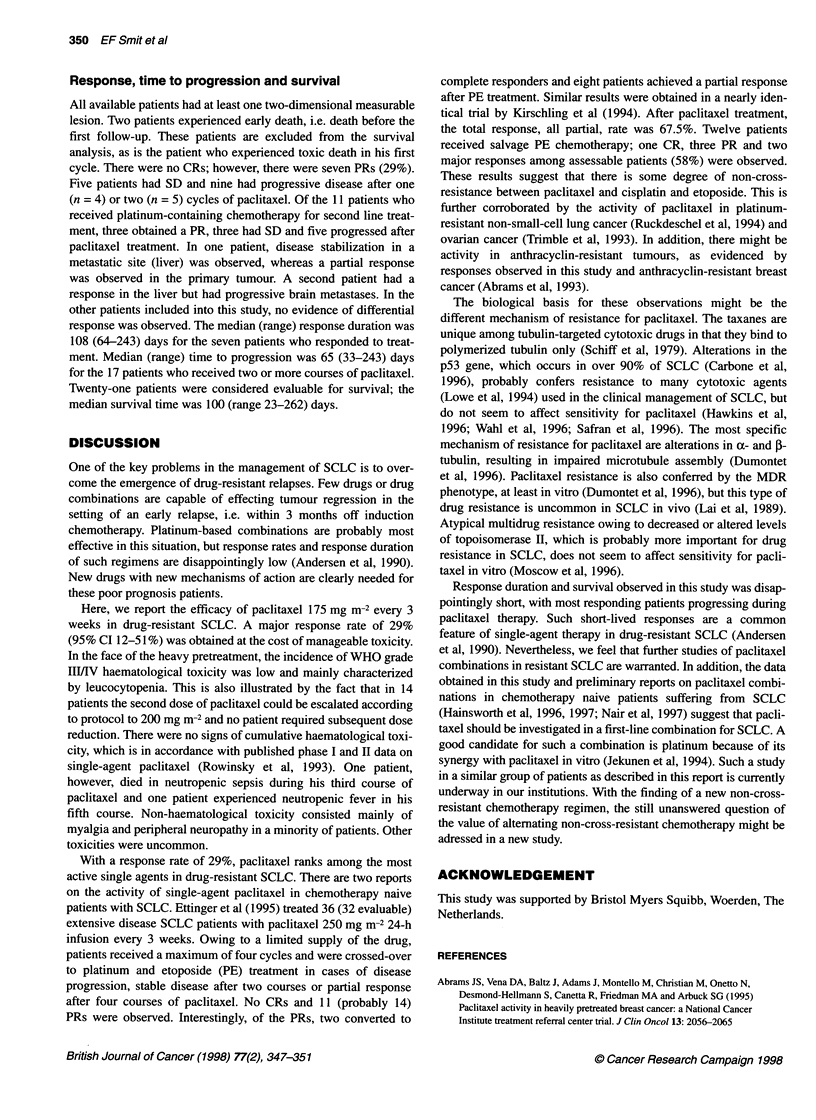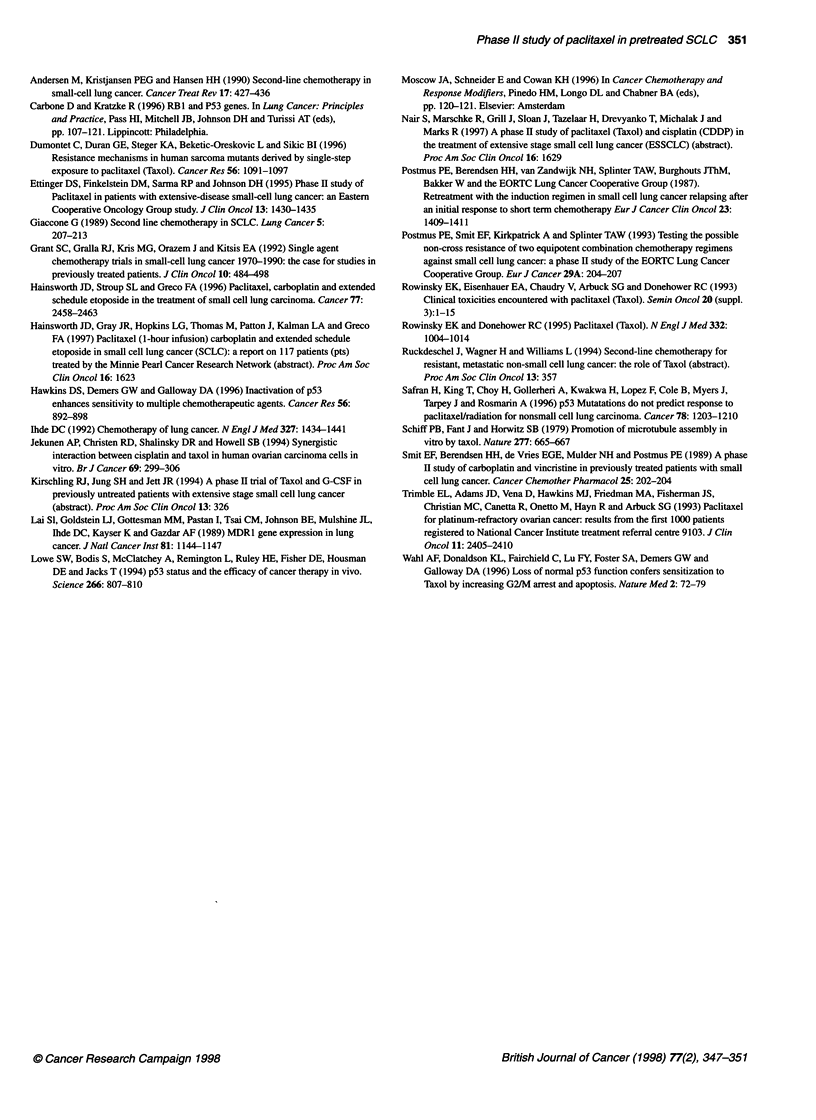# A phase II study of paclitaxel in heavily pretreated patients with small-cell lung cancer.

**DOI:** 10.1038/bjc.1998.54

**Published:** 1998

**Authors:** E. F. Smit, E. Fokkema, B. Biesma, H. J. Groen, W. Snoek, P. E. Postmus

**Affiliations:** Department of Pulmonary Diseases, University Hospital Groningen, The Netherlands.

## Abstract

The purpose of the study was to delineate the efficacy and toxicity of paclitaxel (Taxol, Bristol Myers Squibb) in the treatment of drug resistant small-cell lung cancer (SCLC). Patients with SCLC relapsing within 3 months of cytotoxic therapy received paclitaxel 175 mg m(-2) intravenously over 3 h every 3 weeks. The dose of paclitaxel was adjusted to the toxicity encountered in the previous cycle. Of 24 patients entered into the study, 24 and 21 were assessable for response and toxicity respectively. There were two early deaths and two toxic deaths. No complete and seven partial responses (29%) (95%CI 12-51%) were observed and five patients had disease stabilization. The median survival (n = 21) was 100 days. Life-threatening toxicity occurred in four patients; in others (non)-haematological toxicity was manageable. Paclitaxel is active in drug-resistant SCLC. Further investigation in combination with other active agents in this poor prognosis group is appropriate.


British Journal of Cancer (1998) 77(2), 347–351
© 1998 Cancer Research Campaign

### A phase II study of paclitaxel in heavily pretreated patients with small-cell lung cancer

**EF Smit[1], E Fokkema[1], B Biesma[2], HJM Groen[1], W Snoek[3] and PE Postmus[2]**

[1]Department of Pulmonary Diseases, University Hospital Groningen; [2]Department of Pulmonary Diseases, University Hospital, Free University Amsterdam; [3]Department of Pulmonary Diseases, Martini Hospital Groningen, The Netherlands

**Summary** The purpose of the study was to delineate the efficacy and toxicity of paclitaxel (Taxol, Bristol Myers Squibb) in the treatment of drug resistant small-cell lung cancer (SCLC). Patients with SCLC relapsing within 3 months of cytotoxic therapy received paclitaxel 175 mg $m^{-2}$ intravenously over 3 h every 3 weeks. The dose of paclitaxel was adjusted to the toxicity encountered in the previous cycle. Of 24 patients entered into the study, 24 and 21 were assessable for response and toxicity respectively. There were two early deaths and two toxic deaths. No complete and seven partial responses (29%) (95%CI 12–51%) were observed and five patients had disease stabilization. The median survival ($n$ = 21) was 100 days. Life-threatening toxicity occurred in four patients; in others (non)-haematological toxicity was manageable. Paclitaxel is active in drug-resistant SCLC. Further investigation in combination with other active agents in this poor prognosis group is appropriate.

**Keywords:** small cell lung cancer; chemotherapy; early relapse

With present-day chemo- and radiotherapy regimens, a major response with considerable prolongation of survival (Ihde, 1992) will be achieved in 90–95% of patients with small-cell lung cancer (SCLC). However, in the majority of these patients the tumour will relapse after a shorter or longer treatment-free period. In this situation, second-line treatment is necessary for adequate palliation. A multifocal relapse will usually lead to treatment with chemotherapy (Andersen et al, 1990). It is then necessary to distinguish between patients with tumours that are sensitive and patients with tumours presumably resistant to cytotoxic agents used in the induction phase (Giaccone, 1989). Patients relapsing within 3 months of induction chemotherapy are considered resistant to the drugs used in the induction regimen (Postmus et al, 1987; 1993; Smit et al, 1989). For such patients, second-line treatment should consist off non-cross-resistant drugs. The poor results currently obtained by second-line chemotherapy support the view that failure to identify real non-cross-resistant agents is the primary reason for lack of success (Andersen et al, 1990). Further evidence for this view comes from the fact that, although in most studies on second-line chemotherapy it is not specifically stated, most therapy-resistant patients responding to a second-line regimen have shown a previous response of short duration to first-line treatment (Smit et al, 1989; Postmus, 1993). Thus, there is a great need for new active agents in the setting of second-line treatment in so-called therapy-resistant patients. Moreover, identification of such agents may lead to the development of more potent first-line regimens.

*Received 16 April 1997*
*Revised 23 June 1997*
*Accepted 18 July 1997*

*Correspondence to:* EF Smit, Department of Pulmonary Diseases, Free University Hospital, De Boelelaan 1117, PO Box 7057, 1007 MB Amsterdam, The Netherlands

Paclitaxel (taxol) is a new drug with established activity in resistant solid tumours, such as platinum-resistant ovarian cancer and anthracylin-resistant breast cancer (Rowinsky et al, 1995). In addition, it has shown considerable anti-tumour activity in chemotherapy naive patients with SCLC (Kirschling et al, 1994; Ettinger et al, 1995). Therefore, we initiated a phase II trial of paclitaxel (taxol) in patients with clinically resistant SCLC, which is the subject of this report.

### PATIENTS AND METHODS

The trial was approved by the local Medical Ethics Committees. All patients gave informed consent before they were enrolled into the study.

### Eligibility criteria

Patients were considered eligible when they met all of the following criteria: age between 18 and 75 years; histologically or cytologically proven SCLC; last cytotoxic treatment less than 3 months before entry; ECOG performance status 0–3; WBC $> 3.0 \times 10^9$ $l^{-1}$; platelet count $> 100 \times 10^9$ $l^{-1}$ (unless lower values were because of bone marrow involvement); creatinine clearance according to the Cockroft method > 60 ml $min^{-1}$; bilirubin level less than 25 μmol $l^{-1}$; and bidimensionally measurable disease. Exclusion criteria included significant cardiac disease, uncontrolled infection and concurrent cytotoxic chemotherapy. Concurrent radiotherapy was allowed for, provided that not all measurable lesions were included in the irradiated field.

### Pretreatment evaluation

Before chemotherapy, each patient was evaluated with a history and physical examination with assessment of perfomance status, a complete blood cell count, liver function tests and serum creatinine, electrocardiogram (ECG), chest radiograph and/or computerized tomography of the chest and other staging procedures as indicated, including ultrasonography of the liver and bone scintigraphy.

### Treatment

Paclitaxel was obtained from Bristol-Myers Squibb, Woerden, The Netherlands as a concentrated sterile solution, 6 mg $ml^{-1}$ in 5 ml ampules in polyoxyethylated castor oil (cremophor EL) 50% and dehydrated alcohol. The full calculated dose of paclitaxel was diluted in a minimum volume of 250 ml and a maximum volume of 1000 ml of dextrose 5% or normal saline. To avoid acute allergic reactions all patients received the following medication: dexamethasone 8 mg orally 12 and 6 h before paclitaxel, clemastine 2 mg intravenously (i.v.) push 30 min before paclitaxel and cimetidine 300 mg i.v. push or ranitidine 50 mg i.v. push 30 min before paclitaxel.

Paclitaxel 175 mg $m^{-2}$ was administered as a 3-h i.v. infusion every 21 days. This paclitaxel dose was chosen in view of the chemotherapeutic and radiotherapeutic pretreatment and the short interval between the last treatment and inclusion into this study. The subsequent dose was modified according to toxicity in the previous course for each individual patient. A dose reduction to 150 mg $m^{-2}$ was applied when the WBC count was $< 1.0 \times 10^9\ l^{-1}$ or platelet counts $< 25 \times 10^9\ l^{-1}$ for more than 1 week or in the case of febrile neutropenia. A dose escalation to 200 mg $m^{-2}$ was applied when WBC count was $> 3.0 \times 10^9\ l^{-1}$ and platelet count $> 75 \times 10^9\ l^{-1}$ during the previous cycle. When WHO grade IV myelotoxicity occurs, at this dose level, a dose of 175 mg $m^{-2}$ was to be administered in the subsequent cycle.

Therapy was administered for a maximum of five cycles. Patients went off-study in cases of disease progression, incomplete haematological recovery 2 weeks after scheduled re-treatment, WHO grade III neuropathy or any other non-haematological toxicity WHO grade IV except alopecia.

### Evaluation during treatment

Before each new administration of paclitaxel a physical examination, assessment of performance status, laboratory tests, ECG and chest radiograph or any other investigation necessary for assessment of response was obtained. A complete blood cell count on days 10 and 14 was obtained between two courses of paclitaxel.

### Response assessment

A complete response was defined as the complete resolution of all signs of known disease for a minimum of 4 weeks. A partial response was defined as a more than 50% reduction in the sum of the products of the largest perpendicular diameters of all measurable lesions for a minimum of 4 weeks. The term stable disease was given to patients who failed to fulfil the criteria for partial response in the absence of disease progression. Disease progression was defined as an increase > 25% in the sum of the products of the largest perpendicular diameters of all measurable lesions or the occurrence of any new lesion.

Response duration, time to progression and survival were measured from the date of initiation of therapy. All patients were considered evaluable for toxicity and response.

**Table 1** Patient characteristics

| | |
|---|---|
| Male/female | 20/4 |
| Age median, range | 56 (39–73) years |
| Disease extent | |
| LD | 9 |
| ED | 15 |
| Initial performance status | |
| 0 | 1 |
| 1 | 14 |
| 2 | 6 |
| 3 | 3 |
| Number of previous chemotherapy regimen | |
| 1 | 9 |
| 2 | 11 |
| ≥3 | 4 |
| Radiotherapy | 11 |
| best response to initial chemotherapy | |
| CR | 8 |
| PR | 13 |
| SD | 2 |
| PD | 1 |
| Median (range) time off cytotoxic therapy (weeks) | 4 (0–12) |
| Number of metastatic sites | |
| 1 | 11 |
| 2 | 11 |
| ≥3 | 2 |
| Number of courses total | 69 |
| Median (range) | 3 (1–5) |
| Number of patients with dose | |
| Escalation | 14 (18 evaluable) |
| Reduction | 0 |

The minimum number of patients accrued in the study was that proposed by Grant et al (1992). Twenty patients were enrolled for the first stage. If none of the original 20 patients responded, the drug was inactive and the study terminated. If four responses were observed in the first group of patients the study was to be terminated. If one to three responses were observed, an additional 15 patients were to be enrolled in the second phase. This design permits the exclusion of a response rate greater than 10.9% at the 90% confidence level if no responses were observed in the first phase. With greater true response rates, the probability of declaring a drug active is 0.82 at a true response rate of 16%.

### RESULTS

Between December 1994 and June 1996, 24 patients with relapsed SCLC were treated with paclitaxel. Table 1 lists patient and disease characteristics. One patient with a mixed adeno/small-cell carcinoma of the lung and one patient with a small-cell carcinoma of the oesophagus with multiple lung metastases were included in this study. Their median age was 56 years, with a range of 39–73 years; 83% were male and all but three patients were ambulatory (ECOG performance status 0–2). The median number of previous chemotherapy regimens administered was two, range 1–3. All patients had received cyclophosphamide, doxorubicin and etoposide combination chemotherapy in the induction phase, and a number of different regimens as second-line chemotherapy. For 11 of the last group of patients, second-line chemotherapy consisted of platinum-containing regimens. Eleven patients received radiotherapy, either to the primary tumour and regional lymph nodes

© Cancer Research Campaign 1998

**Table 2** Toxicity of paclitaxel treatment

| A | Haematological leucocytes | *Number of courses associated with WHO grade | |
|---|---|---|---|
| | | Platelets | Haemoglobin |
| 0 | 32 | 54 | 55 |
| I | 16 | 3 | 2 |
| II | 5 | 1 | 4 |
| III | 9 | 3 | 1 |
| IV | 1 | 2 | 1 |

*Two patients not evaluable (early death), four cycles not evaluable.

| B | Non-haematological | *Number of courses associated with WHO grade | | |
|---|---|---|---|---|
| | Nausea/vomiting | Neuropathy | Myalgia** | Oedema** |
| I | 2 | 17 | 10 | 2 |
| II | 4 | 1 | 13 | 0 |
| III | 1*** | 0 | 0 | 0 |

*Two patients not evaluable (early death), four cycles not evaluable. **CTC grading; ***during development of brain metastases.

**Table 3** Response, time to progression and survival

| | Number of patients |
|---|---|
| Complete response | 0 |
| Partial response | 7 (29%; 95% CI 12–51%) |
| Stable disease | 5 |
| Progressive disease | 9 |
| Early death | 2 |
| Toxic death | 1 |
| Response duration | Median (range) days 108 (64–243) |
| Time to progression* | Median (range) days 65 (33–243) |
| Survival** | Median (range) days 100 (23–262) |

*Seven patients non-evaluable (three patients with disease progression after one course; two ED and one TD); **three patients non-evaluable (two ED, one TD).

($n$ = 8) or towards brain metastases ($n$ = 3). The total number of paclitaxel cycles administered was 69, median 3. In 14 patients it was possible to escalate the second dose of paclitaxel, although dose reductions as specified by the protocol were not necessary. In one patient, a single cycle of paclitaxel had to be delayed for 7 days because of incomplete haematological recovery on day 21. All other cycles of paclitaxel were administered as scheduled.

### Toxicity

Complications of treatment were reported according to WHO criteria. In case these criteria did not apply, common toxicity criteria were used. Table 2 lists the incidence of haematological and non-haematological toxicities. Two patients were excluded from this analysis because of rapid disease progression; i.e. before the first evaluation on day 7; in addition, data from four cycles were incomplete. The predominant haematological toxicity was non-cumulative leucocytopenia. WHO grade III/IV leucocytopenia was observed in 10 out of 63 evaluable cycles. In addition, 5 out of 63 evaluable cycles were associated with WHO grade III/IV thrombocytopenia. In four patients, life-threatening complications were encountered. One patient experienced febrile neutropenia during his fifth cycle of paclitaxel, which resolved on antibiotic treatment. One patient with disease progression developed septic shock without leucocytopenia or respiratory failure because of lung oedema on day 16 of cycle 2 and was placed on a ventilator. He was weaned and died at home 2 weeks later because of tumour progression. A third patient, 6 days after his third dose of paclitaxel, was hospitalized with fever, leucocytopenia ($0.2 \times 10^9 l^{-1}$), thrombocytopenia ($7 \times 10^9 l^{-1}$) and elevated liver function tests. Despite extensive supportive measures, this patient died, although in remission, on the second day after hospital admission. The fourth patient with a history of cancer-associated thromboembolism requiring coumarine therapy had an upper gastrointestinal bleeding on day 5 of cycle 1. His platelet counts were normal but the bleeding time was > 200 s. Six days after this episode he received a nasal tube and died the next day, presumably because of massive aspiration while suffering from leuco- and thrombocytopenia.

Non-haematological toxicity was predominantly peripheral neuropathy. Transient myalgia required medication in 13 out of 63 evaluable cycles. Hypersensitivity reactions were not encountered, whereas two patients experienced short-lived peripheral oedema.

© Cancer Research Campaign 1998

### Response, time to progression and survival

All available patients had at least one two-dimensional measurable lesion. Two patients experienced early death, i.e. death before the first follow-up. These patients are excluded from the survival analysis, as is the patient who experienced toxic death in his first cycle. There were no CRs; however, there were seven PRs (29%). Five patients had SD and nine had progressive disease after one ($n = 4$) or two ($n = 5$) cycles of paclitaxel. Of the 11 patients who received platinum-containing chemotherapy for second line treatment, three obtained a PR, three had SD and five progressed after paclitaxel treatment. In one patient, disease stabilization in a metastatic site (liver) was observed, whereas a partial response was observed in the primary tumour. A second patient had a response in the liver but had progressive brain metastases. In the other patients included into this study, no evidence of differential response was observed. The median (range) response duration was 108 (64–243) days for the seven patients who responded to treatment. Median (range) time to progression was 65 (33–243) days for the 17 patients who received two or more courses of paclitaxel. Twenty-one patients were considered evaluable for survival; the median survival time was 100 (range 23–262) days.

### DISCUSSION

One of the key problems in the management of SCLC is to overcome the emergence of drug-resistant relapses. Few drugs or drug combinations are capable of effecting tumour regression in the setting of an early relapse, i.e. within 3 months off induction chemotherapy. Platinum-based combinations are probably most effective in this situation, but response rates and response duration of such regimens are disappointingly low (Andersen et al, 1990). New drugs with new mechanisms of action are clearly needed for these poor prognosis patients.

Here, we report the efficacy of paclitaxel 175 mg $m^{-2}$ every 3 weeks in drug-resistant SCLC. A major response rate of 29% (95% CI 12–51%) was obtained at the cost of manageable toxicity. In the face of the heavy pretreatment, the incidence of WHO grade III/IV haematological toxicity was low and mainly characterized by leucocytopenia. This is also illustrated by the fact that in 14 patients the second dose of paclitaxel could be escalated according to protocol to 200 mg $m^{-2}$ and no patient required subsequent dose reduction. There were no signs of cumulative haematological toxicity, which is in accordance with published phase I and II data on single-agent paclitaxel (Rowinsky et al, 1993). One patient, however, died in neutropenic sepsis during his third course of paclitaxel and one patient experienced neutropenic fever in his fifth course. Non-haematological toxicity consisted mainly of myalgia and peripheral neuropathy in a minority of patients. Other toxicities were uncommon.

With a response rate of 29%, paclitaxel ranks among the most active single agents in drug-resistant SCLC. There are two reports on the activity of single-agent paclitaxel in chemotherapy naive patients with SCLC. Ettinger et al (1995) treated 36 (32 evaluable) extensive disease SCLC patients with paclitaxel 250 mg $m^{-2}$ 24-h infusion every 3 weeks. Owing to a limited supply of the drug, patients received a maximum of four cycles and were crossed-over to platinum and etoposide (PE) treatment in cases of disease progression, stable disease after two courses or partial response after four courses of paclitaxel. No CRs and 11 (probably 14) PRs were observed. Interestingly, of the PRs, two converted to complete responders and eight patients achieved a partial response after PE treatment. Similar results were obtained in a nearly identical trial by Kirschling et al (1994). After paclitaxel treatment, the total response, all partial, rate was 67.5%. Twelve patients received salvage PE chemotherapy; one CR, three PR and two major responses among assessable patients (58%) were observed. These results suggest that there is some degree of non-cross-resistance between paclitaxel and cisplatin and etoposide. This is further corroborated by the activity of paclitaxel in platinum-resistant non-small-cell lung cancer (Ruckdeschel et al, 1994) and ovarian cancer (Trimble et al, 1993). In addition, there might be activity in anthracyclin-resistant tumours, as evidenced by responses observed in this study and anthracyclin-resistant breast cancer (Abrams et al, 1993).

The biological basis for these observations might be the different mechanism of resistance for paclitaxel. The taxanes are unique among tubulin-targeted cytotoxic drugs in that they bind to polymerized tubulin only (Schiff et al, 1979). Alterations in the p53 gene, which occurs in over 90% of SCLC (Carbone et al, 1996), probably confers resistance to many cytotoxic agents (Lowe et al, 1994) used in the clinical management of SCLC, but do not seem to affect sensitivity for paclitaxel (Hawkins et al, 1996; Wahl et al, 1996; Safran et al, 1996). The most specific mechanism of resistance for paclitaxel are alterations in $\alpha$- and $\beta$-tubulin, resulting in impaired microtubule assembly (Dumontet et al, 1996). Paclitaxel resistance is also conferred by the MDR phenotype, at least in vitro (Dumontet et al, 1996), but this type of drug resistance is uncommon in SCLC in vivo (Lai et al, 1989). Atypical multidrug resistance owing to decreased or altered levels of topoisomerase II, which is probably more important for drug resistance in SCLC, does not seem to affect sensitivity for paclitaxel in vitro (Moscow et al, 1996).

Response duration and survival observed in this study was disappointingly short, with most responding patients progressing during paclitaxel therapy. Such short-lived responses are a common feature of single-agent therapy in drug-resistant SCLC (Andersen et al, 1990). Nevertheless, we feel that further studies of paclitaxel combinations in resistant SCLC are warranted. In addition, the data obtained in this study and preliminary reports on paclitaxel combinations in chemotherapy naive patients suffering from SCLC (Hainsworth et al, 1996, 1997; Nair et al, 1997) suggest that paclitaxel should be investigated in a first-line combination for SCLC. A good candidate for such a combination is platinum because of its synergy with paclitaxel in vitro (Jekunen et al, 1994). Such a study in a similar group of patients as described in this report is currently underway in our institutions. With the finding of a new non-cross-resistant chemotherapy regimen, the still unanswered question of the value of alternating non-cross-resistant chemotherapy might be adressed in a new study.

### ACKNOWLEDGEMENT

This study was supported by Bristol Myers Squibb, Woerden, The Netherlands.

### REFERENCES

Abrams JS, Vena DA, Baltz J, Adams J, Montello M, Christian M, Onetto N, Desmond-Hellmann S, Canetta R, Friedman MA and Arbuck SG (1995) Paclitaxel activity in heavily pretreated breast cancer: a National Cancer Institute treatment referral center trial. *J Clin Oncol* **13**: 2056–2065

© Cancer Research Campaign 1998

Andersen M, Kristjansen PEG and Hansen HH (1990) Second-line chemotherapy in small-cell lung cancer. *Cancer Treat Rev* **17**: 427–436

Carbone D and Kratzke R (1996) RB1 and P53 genes. In *Lung Cancer: Principles and Practice*, Pass HI, Mitchell JB, Johnson DH and Turissi AT (eds), pp. 107–121. Lippincott: Philadelphia.

Dumontet C, Duran GE, Steger KA, Beketic-Oreskovic L and Sikic BI (1996) Resistance mechanisms in human sarcoma mutants derived by single-step exposure to paclitaxel (Taxol). *Cancer Res* **56**: 1091–1097

Ettinger DS, Finkelstein DM, Sarma RP and Johnson DH (1995) Phase II study of Paclitaxel in patients with extensive-disease small-cell lung cancer: an Eastern Cooperative Oncology Group study. *J Clin Oncol* **13**: 1430–1435

Giaccone G (1989) Second line chemotherapy in SCLC. *Lung Cancer* **5**: 207–213

Grant SC, Gralla RJ, Kris MG, Orazem J and Kitsis EA (1992) Single agent chemotherapy trials in small-cell lung cancer 1970–1990: the case for studies in previously treated patients. *J Clin Oncol* **10**: 484–498

Hainsworth JD, Stroup SL and Greco FA (1996) Paclitaxel, carboplatin and extended schedule etoposide in the treatment of small cell lung carcinoma. *Cancer* **77**: 2458–2463

Hainsworth JD, Gray JR, Hopkins LG, Thomas M, Patton J, Kalman LA and Greco FA (1997) Paclitaxel (1-hour infusion) carboplatin and extended schedule etoposide in small cell lung cancer (SCLC): a report on 117 patients (pts) treated by the Minnie Pearl Cancer Research Network (abstract). *Proc Am Soc Clin Oncol* **16**: 1623

Hawkins DS, Demers GW and Galloway DA (1996) Inactivation of p53 enhances sensitivity to multiple chemotherapeutic agents. *Cancer Res* **56**: 892–898

Ihde DC (1992) Chemotherapy of lung cancer. *N Engl J Med* **327**: 1434–1441

Jekunen AP, Christen RD, Shalinsky DR and Howell SB (1994) Synergistic interaction between cisplatin and taxol in human ovarian carcinoma cells in vitro. *Br J Cancer* **69**: 299–306

Kirschling RJ, Jung SH and Jett JR (1994) A phase II trial of Taxol and G-CSF in previously untreated patients with extensive stage small cell lung cancer (abstract). *Proc Am Soc Clin Oncol* **13**: 326

Lai SI, Goldstein LJ, Gottesman MM, Pastan I, Tsai CM, Johnson BE, Mulshine JL, Ihde DC, Kayser K and Gazdar AF (1989) MDR1 gene expression in lung cancer. *J Natl Cancer Inst* **81**: 1144–1147

Lowe SW, Bodis S, McClatchey A, Remington L, Ruley HE, Fisher DE, Housman DE and Jacks T (1994) p53 status and the efficacy of cancer therapy in vivo. *Science* **266**: 807–810

Moscow JA, Schneider E and Cowan KH (1996) In *Cancer Chemotherapy and Response Modifiers*, Pinedo HM, Longo DL and Chabner BA (eds), pp. 120–121. Elsevier: Amsterdam

Nair S, Marschke R, Grill J, Sloan J, Tazelaar H, Drevyanko T, Michalak J and Marks R (1997) A phase II study of paclitaxel (Taxol) and cisplatin (CDDP) in the treatment of extensive stage small cell lung cancer (ESSCLC) (abstract). *Proc Am Soc Clin Oncol* **16**: 1629

Postmus PE, Berendsen HH, van Zandwijk NH, Splinter TAW, Burghouts JThM, Bakker W and the EORTC Lung Cancer Cooperative Group (1987). Retreatment with the induction regimen in small cell lung cancer relapsing after an initial response to short term chemotherapy *Eur J Cancer Clin Oncol* **23**: 1409–1411

Postmus PE, Smit EF, Kirkpatrick A and Splinter TAW (1993) Testing the possible non-cross resistance of two equipotent combination chemotherapy regimens against small cell lung cancer: a phase II study of the EORTC Lung Cancer Cooperative Group. *Eur J Cancer* **29A**: 204–207

Rowinsky EK, Eisenhauer EA, Chaudry V, Arbuck SG and Donehower RC (1993) Clinical toxicities encountered with paclitaxel (Taxol). *Semin Oncol* **20** (suppl. 3):1–15

Rowinsky EK and Donehower RC (1995) Paclitaxel (Taxol). *N Engl J Med* **332**: 1004–1014

Ruckdeschel J, Wagner H and Williams L (1994) Second-line chemotherapy for resistant, metastatic non-small cell lung cancer: the role of Taxol (abstract). *Proc Am Soc Clin Oncol* **13**: 357

Safran H, King T, Choy H, Gollerheri A, Kwakwa H, Lopez F, Cole B, Myers J, Tarpey J and Rosmarin A (1996) p53 Mutatations do not predict response to paclitaxel/radiation for nonsmall cell lung carcinoma. *Cancer* **78**: 1203–1210

Schiff PB, Fant J and Horwitz SB (1979) Promotion of microtubule assembly in vitro by taxol. *Nature* **277**: 665–667

Smit EF, Berendsen HH, de Vries EGE, Mulder NH and Postmus PE (1989) A phase II study of carboplatin and vincristine in previously treated patients with small cell lung cancer. *Cancer Chemother Pharmacol* **25**: 202–204

Trimble EL, Adams JD, Vena D, Hawkins MJ, Friedman MA, Fisherman JS, Christian MC, Canetta R, Onetto M, Hayn R and Arbuck SG (1993) Paclitaxel for platinum-refractory ovarian cancer: results from the first 1000 patients registered to National Cancer Institute treatment referral centre 9103. *J Clin Oncol* **11**: 2405–2410

Wahl AF, Donaldson KL, Fairchield C, Lu FY, Foster SA, Demers GW and Galloway DA (1996) Loss of normal p53 function confers sensitization to Taxol by increasing G2/M arrest and apoptosis. *Nature Med* **2**: 72–79

© Cancer Research Campaign 1998